# Inferring Authors’ Relative Contributions to Publications from the Order of Their Names When Default Order Is Alphabetical

**DOI:** 10.3390/e22090927

**Published:** 2020-08-24

**Authors:** Yigal Gerchak

**Affiliations:** Department of Industrial Engineering, Tel Aviv University, Tel-Aviv 69978, Israel; yigal@tauex.tau.ac.il

**Keywords:** OR in Scientometrics, joint authorship, apportioning credit, maximum entropy

## Abstract

In attributing individual credit for co-authored academic publications, one issue is how to *apportion* (unequal) credit, based on the order of authorship. Apportioning credit for completed joint undertakings has always been a challenge. Academic promotion committees are faced with such tasks regularly, when trying to infer a candidate’s contribution to an article they coauthored with others. We propose a method for achieving this goal in disciplines (such as the author’s) where the default order is alphabetical. The credits are those maximizing Shannon entropy subject to order constraints.

## 1. Introduction

More and more published research is a collaboration of several researchers (Shapiro et al., 1994 [1]). As various promotion committees need to know and estimate the contribution and the quality of an individual researcher, that raises the bibliometric issue of apportioning individual credit by the “fractional counting“ of joint publications (e.g., references [2,3,4]). Abbas (2011) [2] proposes a set of indices to evaluate the quality of research produced by an author, while Egghe (2008) [4] focuses on a mathematical theory of the h- and g-index in the case of the fractional counting of authorship.

In this paper, we focus on disciplines where the default order of authors is alphabetical (e.g., Social Science and Mathematics; Liu and Fang 2014 [5]). Thus, for example, an order such as (B, A, C) indicates that B’s contribution was “significantly” larger than A’s, while C’s was not. We shall thus assume that each discipline has a “standard”, where if, for example, B’s contribution exceeds A’s by more than the standard, their order will be switched. Therefore, the default order (A, B, C) indicates that neither B’s nor C’s excess contribution exceeds the standard. Other than these inferences, which become constraints on the fractional contributions, we shall assume that the contributions are as uncertain as possible.

Suppose there is a disciplinary standard ε, 0<ε<1, such that the alphabetical order is *not* changed unless the difference in contributions, in favor of the alphabetically latter author, is deemed to be larger than ε. Thus, if, for example, the order of three authors is (B, A, C) (meaning that the author who is alphabetically second is listed before the one who is alphabetically first), it reflects the fact that B’s contribution exceeds A’s by more than ε, while C’s contribution does not exceed A’s (and thus also not B’s) by more than ε.  A large value of ε indicates strong adherence to an alphabetical order, while small values correspond to a high sensitivity to the actual relative contributions. The standard ε  may or may not be known to those wishing to evaluate the contributions.

We shall make use of the constrained *maximal* (Shannon) entropy approach, reflecting the most diffused contribution distribution that satisfies the implications of the limited information given by the order. Constrained maximal entropy has been used, among other applications, in physics [6] and finance [7], where the constraints were the mean and/or variance of the distribution. Our constraints are simpler, so solving the problem is often rather trivial. First, we deal with estimating the mean contribution, and then, we propose an appropriate multivariate distribution.

## 2. Mean Contribution

Start with two authors (A and B), with the respective unknown expected contributions p for A and 1−p for B, which we wish to infer. Note that although p is a share and not a probability, we shall perform probability operations on it. The entropy function,

−p log $p-\left(1-p\right)$ log $\left(1-p\right)$, is concave in p with its maximum at $p=\frac{1}{2}$, regardless of the base of the logarithm (e.g., Cover and Thomas 2006 [8]). We shall assume that each author’s contribution is at least $\delta \left(\delta <\frac{1}{4}\right).$Now, the order (A, B) implies that p>1−p−ε, i.e., that $p>\frac{1-\varepsilon }{2}$. Since $\frac{1-\varepsilon }{2}<\frac{1}{2}\forall \varepsilon ,$ it follows that the constrained entropy is maximized at $p^{*}=\frac{1}{2}.$ Thus, the authors are deemed to have contributed equally(!). The order (B, A) implies that 1−p>p+ε, i.e., that

$p<\frac{1-\varepsilon }{2}<\frac{1}{2}$, so $p^{*}=\frac{1-\varepsilon }{2},\;\varepsilon <1$. If $\frac{1}{2}-\frac{1}{2}\varepsilon <\delta$, then p=δ. Thus, if, for example, $\varepsilon =\frac{1}{4}$, A’s mean contribution is estimated at $\frac{3}{8}$. If $\varepsilon =\frac{1}{4}$ A’s mean contribution is $max\left(\frac{1}{8},\delta \right)$, where the low value reflects A’s demotion despite the high threshold.

For three authors A, B and C, and respective unknown mean shares p, q, 1−p−q, the unconstrained entropy is jointly concave and maximized at $\left(\frac{1}{3},\;\frac{1}{3},\;\frac{1}{3}\right)$. Now, if the order is (A, B, C), it follows that p>q−ε and q>1−p−q−ε. The feasible solution with the highest entropy is $\left(\frac{1}{3}-\frac{\varepsilon }{2},\;\frac{1}{3},\;\frac{1}{3}+\frac{\varepsilon }{2}\right)$. If the order is (B, A, C), the constraints are q>p+ε, q>1−p−q−ε and 0≤p+q≤1. Among the feasible solutions, the one that maximizes the entropy is $\frac{1}{3}-\frac{2}{2}\varepsilon \;,\;\frac{1}{3}+\frac{1}{3}\varepsilon ,\;\frac{1}{3}+\frac{1}{3}\varepsilon \;,$ assuming $\varepsilon <\frac{1}{2}$ (note that our notation lists the relative contributions in the alphabetical order of the authors).

Note that *C’s* contribution, $\frac{1}{3}+\frac{1}{3}\varepsilon$, is deemed to be larger that A’s $\left(\frac{1}{3}-\frac{2}{3}\varepsilon \right)$, even though they appear later in the order (but not by more than 2ε).

For (*B, C, A*), the solution is $\frac{1}{3}-\frac{2}{3}\varepsilon ,\;\frac{1}{3}+\frac{1}{3}\varepsilon ,\;\frac{1}{\;3}+\frac{1}{3}\varepsilon ,\;\varepsilon <\frac{1}{2}$. If $\frac{1}{3}-\frac{2}{3}\varepsilon <\delta ,$ then we have $\left\{\delta ,\delta +\varepsilon ,\;\delta +\varepsilon \right\}.$ Note that if ε is large, then as A was nevertheless demoted to last, its contribution is deemed to be negligible (another school of thought is that if all the authors were essential to the research, they should receive equal credit. If one adopts the philosophy that all the authors were essential to the creation of the paper, a possible approach would be to *average* the above order-dependent shares with equal shares. Thus, for example, the order (B, C, A) will result in $\frac{1}{2}\left\{\left(\frac{1}{3}-\varepsilon ,\;\frac{1}{3}+\frac{\varepsilon }{2},\;\frac{1}{3}+\frac{\varepsilon }{2}\right)+\left(\frac{1}{3},\;\frac{1}{3},\;\frac{1}{3}\right)\right\}=\frac{1}{3}-\frac{\varepsilon }{2},\;\frac{1}{3}+\frac{\varepsilon }{4},\;\frac{1}{3}+\frac{\varepsilon }{4},\;\varepsilon <\frac{2}{3}$).

For (A, C, B), the solutions is $\frac{1}{3}-\frac{1}{3}\varepsilon ,\;\frac{1}{3}-\frac{1}{3}\varepsilon ,\;\frac{1}{3}+\frac{2}{3}\varepsilon ,\;\varepsilon <1$. If $\frac{1}{3}-\frac{1}{3}\varepsilon <\delta$, then $\left\{\delta ,\;\delta ,\;1-2\delta \right\}.$ If ε<1, then the allocation is δ,δ, 1−2δ, so A’s and B’s contribution is deemed negligible. The intuition here is that despite the high requirement for reversing an order $\left(\varepsilon >1\right)$, C has overtaken B.

For (C, B, A), the solution is $\left(\frac{1}{3}-\varepsilon ,\;\frac{1}{3},\frac{1}{3}+\varepsilon \right)$, ε+δ<1.

Consider now the case of *four* authors, whose mean contributions p, q, r, 1−p−q−r we wish to find.

For (A, B, C, D), the mean contributions need to satisfy

$$q-p<\varepsilon^{\;}$$

$$r-q<\varepsilon^{\;}$$

$$r-q<\varepsilon^{\;}$$

$$1-p-q-r-r<\varepsilon^{\;}$$

$$\left(\Rightarrow r>\frac{1-p-q-\varepsilon }{2}\right)$$

⇒ Max entropy is attained at

$$\frac{1}{4}-\frac{3}{2}\varepsilon ,\;\frac{1}{4}-\frac{1}{2}\varepsilon ,\;\frac{1}{4}+\frac{1}{2}\varepsilon ,\;\frac{1}{4}+\frac{3}{2}\varepsilon ,\varepsilon <\frac{1}{6}$$

For (D, C, B, A), the mean contributions need to satisfy

1−p−q−r>r+ε

$$\left(\Rightarrow r<\frac{1-p-q-\varepsilon }{2}\right)$$

r>q+ε

p<q−ε

⇒ Max entropy is attained at

$$\frac{1}{4}-\frac{3}{2}\varepsilon ,\;\frac{1}{4}-\frac{1}{2}\varepsilon ,\;\frac{1}{4}+\frac{1}{2}\varepsilon ,\;\frac{1}{4}+\frac{3}{2}\varepsilon ,\;\varepsilon <\frac{1}{6}.$$

If $\varepsilon >\frac{1}{6}$, then the allocation is $\left(0,\;\frac{1}{6},\;\frac{1}{3},\;\frac{1}{2}\;\right)$. For (A, B, D, C), we obtain $\left(\frac{1}{4}-\varepsilon ,\;\frac{1}{\;4},\;\frac{1}{4},\;\frac{1}{4}+\varepsilon \right)$, and so forth.

Note that in all cases, the expected contributions are either independent of ε or dependent on it linearly. Thus, if ε  is a random variable (with some subjective distribution), the only change required is the substitution of $E\left(\varepsilon \right)$ for ε wherever it appears.

## 3. Joint Distribution of Relative Contributions

We shall now assume that the joint distribution of the relative contributions is believed to be *Dirichlet* (e.g., Kotz, Balakrishnan and Johnson 2000, 40.1 [9]). That is, the joint density of the relative contributions $p_{1},\ldots ,p_{m}$ is

$$f_{p_{1},\ldots ,p_{m}}\left(p_{1},\ldots ,p_{m}\right)=\frac{\Gamma \left(\sum_{j=0}^{m}\theta_{j}\right)}{\prod_{j=1}^{m}\Gamma \left(\theta_{j}\right)}{\left(1-\sum_{j=1}^{m}p_{j}\right)}^{\theta_{0}-1}\prod_{j=1}^{m}p_{j}^{\theta_{j}-1},\;\;\;p_{j}\geq 0,\;\;j=1,\ldots ,m,\;\;\;\sum_{j=1}^{m}p_{j}\leq 1.$$

We have $\theta_{0}=1$, so $E\left(p_{i}\right)=\frac{\theta_{i}}{1+\sum_{j=1}^{m}\theta_{j}},\;i=1,\ldots ,m$. Note that the marginal density of $p_{i}$ is beta $\left(\theta_{i},\;\overset{m}{\underset{j=0}{\sum }}\theta_{j}-\theta_{i}\right)$, so $E\left(p_{i}\right)=\frac{\theta_{i}}{\sum_{j=0}^{m}\theta_{j}}$,

and

$$\mathrm{Var}\;\left(p_{i}\right)=\frac{\theta_{i}\left(\sum_{j=0}^{m}\theta_{j}-\theta_{i}\right)}{{\left(\sum_{j=1}^{m}\theta_{j}\right)}^{2}\left(\sum_{j=1}^{m}\theta_{j}+1\right)}$$

and

$$\mathrm{corr}\;\left(p_{i},\;p_{j}\right)=-\sqrt{\frac{\theta_{i}\theta_{j}}{\left(\sum_{k=0}^{m}\theta_{k}-\theta_{i}\right)\left(\sum_{k=0}^{m}\theta_{k}-\theta_{j}\right)}}.$$

As we wish to allocate the whole credit to the authors, we have $\theta_{0}=1$,

So $E\left(p_{i}\right)=\frac{\theta_{i}}{1+\sum_{j=1}^{m}\theta_{j}},\;i=1,\ldots ,m$.

Note that if we have already estimated (inferred) the mean relative contributions $p_{1},\ldots ,p_{m},$ then, to maintain these ratios, we need to have $\theta_{i}=kp_{i},\;i=1,\ldots ,m$, for some k>0. The choice of k will determine the parameters of the (Dirichlet) distribution.

## 4. Effect of Number of Authors

How does the number of authors affect the relative contribution of one of them? Consider, for concreteness, A’s contribution in orders where he/she is last:

$$2.\;BA\to \frac{1}{2}-\frac{\varepsilon }{2}\;,\;\varepsilon <1.$$

3a. CBA→δ

$$3b.\;BCA\to \frac{1}{3}-\varepsilon ,\;\varepsilon <\frac{1}{3}$$

We see that, in the case of three authors, A’s relative contribution depends on the order of B and C; in 3a, C needs to be rewarded for “overtaking “ B (as well as A), which reduces A’s contributions.

$$4a.\;DCBA\to \frac{1}{4}-\frac{3}{2}\varepsilon ,\;\varepsilon <\frac{1}{6}$$

$$4b.\;BCDA\to \frac{1}{4}-3\varepsilon ,\;\varepsilon <\frac{1}{12\;}$$

Now, for 3b and 4a, the relative contribution of A is:

$$\frac{BA=\frac{1}{2}-\frac{\varepsilon }{2}}{BCA=\frac{1}{3}-\varepsilon }=\frac{3\left(1-\varepsilon \right)}{2\left(1-3\varepsilon \right)},\;\frac{BCA=\frac{1}{3}-\varepsilon }{DCBA=\frac{1}{4}-\frac{3}{2}\varepsilon }=\frac{4\left(1-3\varepsilon \right)}{3\left(1-6\varepsilon \right)}.$$

The former ratio is larger than the latter for, and only for, $\varepsilon <\frac{-15+\sqrt{294}}{36}\approx 0.07$. Thus, no general conclusion is possible.

For 3a and 4a, $\frac{BA}{BCA}$ is larger if $\varepsilon >\frac{7+\sqrt{25+18}\delta^{2}}{12}$.

For 4b, the former ratio is larger if $1<\varepsilon <\frac{13}{12}$.

For *n* authors in alphabetical internal order with A at the end, A’s contribution is $\frac{1}{n}-\frac{n-1}{2}\varepsilon ,\;\varepsilon <\frac{2}{\left(n-1\right)n}$. If $\varepsilon >\frac{2}{\left(n-1\right)n\;}$, A’s contribution is δ.

## 5. Short Discussion and Concluding Remarks

In this paper, we attempted to quantify the significance of deviations from some “natural“ or “default“ order of authors. We assumed that the unknown relative contributions maximize entropy subject to constraints reflecting default order reversal.

Future study could further demonstrate and validate the proposed method by empirical and data-driven methods. For example, a comparison could be made of academics’ rankings by the H-index (or some other measures), compared to authors’ rankings by their total fractional journal paper contributions. Another option is to apply this study to journals where the authors’ contributions are required and published, while comparing it to the order of the authors’ names. Clearly, a survey could be conducted over some well-cited papers, asking the authors to ascribe their fractional contributions, while comparing them to those determined by the proposed method.

We note that the proposed measure, if and when it will be used for personal evaluation and promotion, should be combined with qualitative assessments, as often done in T&P committees. Otherwise, “automated” evaluation metrics alone could encourage game-playing among collaborators, which would be an uninvited outcome.

Finally, let us note that several related applications could use, with required modifications, the proposed method—for example, for assessing the contribution of programmers and AI agents in tasks distributed and published over the Internet. One scenario with some similarity to the considered problem is the following.

In sports, some media “power rank” (PR) teams during the season. The PR is usually consistent with the number of wins the teams have achieved, but not always (there might be other factors such as recent injuries, recent performance, etc.).

Therefore, suppose that the number of wins of two teams A and B $\left(n,m\right)$ are such that n=m+1, while team B is power ranked before A. Thus, $1-p>p+\varepsilon \Rightarrow p^{*}=\frac{1}{2}-\frac{1}{2}\varepsilon$.

If $n=m+2,\;1-p>p+2\varepsilon \Rightarrow p^{*}=\frac{1}{2}-\varepsilon$.
